
JOURNAL INFORMATION
==============================
NLM Title Abbreviation: Brain Behav
Journal ID: Brain Behav
NLM Journal ID: 101570837
Journal ID: brb3

Brain and Behavior

EISSN: 2162-3279
Publisher: Wiley

ARTICLE INFORMATION
==============================
PMCID: PMC4055182
DOI: 10.1002/brb3.166
Article version: 1
Subjects: Issue Information

Issue Information

Print publication date: 2014 May
Electronic publication date: 2014 May 14
Volume: 4
Issue: 3
First page: i
Last page: iii
Copyright: © 2014 Brain and Behavior published by Wiley Periodicals, Inc.
Copyright year: 2014
License: This is an open access article under the terms of the Creative Commons Attribution License, which permits use, distribution and reproduction in any medium, provided the original work is properly cited.
License URL: https://creativecommons.org/licenses/by/3.0/

==============================
Volume 4, Number 3

### Brain and Behavior

Open Access

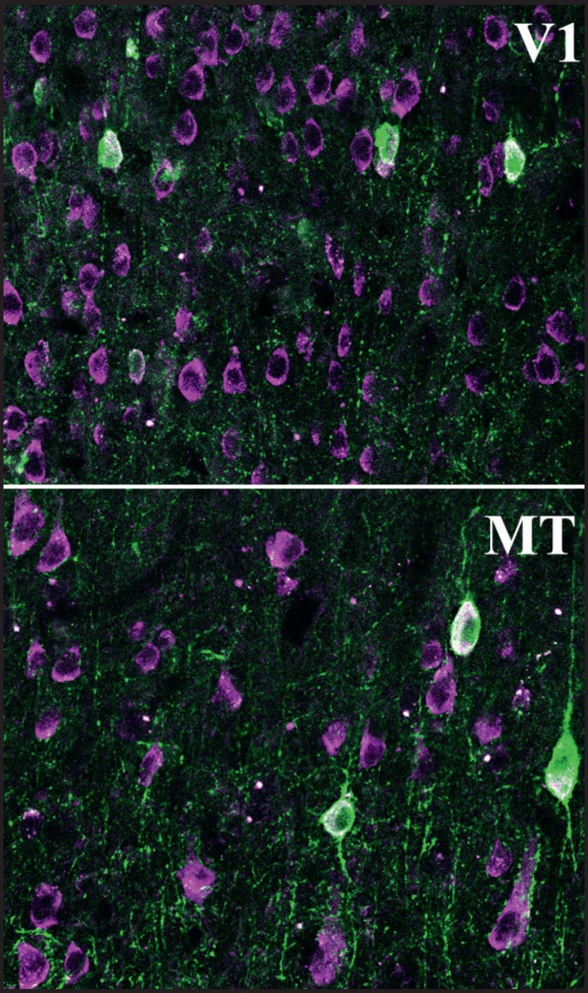

Editors-in-Chief
Andrei V. Alexandrov
Maryann E. Martone

WILEY

Brain and Behavior

Open Access

### Aims and Scope

*Brain and Behavior* is a peer-reviewed, interdisciplinary journal, providing rapid publication of high-quality research across neurology, neuroscience, psychology and psychiatry. The journal will publish quality research reports that enhance understanding of the brain and behavior.

*Brain and Behavior* will give rapid consideration to papers in all areas of clinical and basic research. Molecular, cellular, systems and population-level research in humans and animal models are welcome. The journal will consider empirical and theoretical submissions in areas including but not limited to mechanisms and treatment of all neurological diseases and psychiatric disorders, behavioral and cognitive science, neuronal and glial cell biology, neurosurgery, neurophysiology, neuropharmacology, neuroimmunology, neuropathology, computational neuroscience, functional and structural neuroimaging, neurogenetics and psychiatric genetics, child and adolescent psychiatry, psychiatry of affective and cognitive disorders, epidemiology, emerging technologies and new research methods, translational research, neuropsychology, clinical psychology, and developmental, cognitive, and social psychology.

*Brain and Behavior* features original research articles, reviews, methods papers, editorials, and commentaries. Original research papers must report well-conducted research with conclusions supported by the data presented in the paper.

*Brain and Behavior* publishes papers submitted directly to the journal and those referred from a select group of prestigious journals published by Wiley-Blackwell. *Brain and Behavior* is a Wiley Open Access journal, one of a new series of peer-reviewed titles publishing quality research with speed and efficiency. For further information visit the Wiley Open Access website at http://www.wileyopenaccess.com.

### Open Access and Copyright

All articles published by *Brain and Behavior* are fully open access: immediately freely available to read, download and share. All articles accepted from 14 August 2012 are published under the terms of the Creative Commons Attribution License. All articles accepted before this date were published under a Creative Commons Attribution Non-Commercial License. The Creative Commons Attribution License permits use, distribution and reproduction in any medium, provided the original work is properly cited and allows the commercial use of published articles.

Copyright on any research article in a journal published by *Brain and Behavior* is retained by the author(s). Authors grant Wiley a license to publish the article and identify itself as the original publisher. Authors also grant any third party the right to use the article freely as long as its integrity is maintained and its original authors, citation details and publisher are identified. Further information about open access license and copyright can be found at http://www.wileyopenaccess.com/details/content/12f25db4c87/Copyright--License.html.

### Purchasing Print Reprints

Print reprints of Wiley Open Access articles can be purchased from corporatesales@wiley.com.

### Disclaimer

The Publisher and Editors cannot be held responsible for errors or any consequences arising from the use of information contained in this journal; the views and opinions expressed do not necessarily reflect those of the Publisher and Editors, neither does the publication of advertisements constitute any endorsements by the Publisher and Editors of the products advertised.

Wiley Open Access articles posted to repositories or websites are without warranty from Wiley of any kind, either express or implied, including, but not limited to, warranties of merchantability, fitness for a particular purpose, or non-infringement. To the fullest extent permitted by law Wiley disclaims all liability for any loss or damage arising out of, or in connection with, the use of or inability to use the content.

*Front cover: Cholinergic targeting of inhibition versus excitation in the visual cortex. Photo reproduced by permission of Anita A. Disney, Systems Neurobiology Laboratories, The Salk Institute for Biological Studies, La Jolla, CA, USA.*

### Editors-in-Chief

Andrei V. Alexandrov, MD
Maryann E. Martone, PhD
*Address correspondence to the Editorial Office: brain@wiley.com*

### Editorial Board

*Associate Editors:*

Dona Lee Wong
*Harvard Medical School, McLean Hospital, USA*

*Editorial Board Members:*

Hubert Amrein
*Texas A&M, USA*

Isabelle Aubert
*University of Toronto, Canada*

David Belin
*University of Poitiers, France*

Sandra Black
*University of Toronto, Canada*

Sandra Bosacki
*Brock University, Canada*

Robert Bryan
*Baylor College of Medicine, USA*

Hans Crombag
*University of Sussex, United Kingdom*

Mirella Dapretto
*University of California, Los Angeles, USA*

David Diamond
*University of South Florida, USA*

Scott Grafton
*University of California, Santa Barbara, USA*

Ilona Grunwald-Kadow
*Max Planck Institute of Neurobiology, Germany*

Martin Hagger
*Curtin University, Australia*

Jozsef Haller
*Hungarian Academy of Sciences, Hungary*

Ahmad Hariri
*Duke University, USA*

Shu-Leong Ho
*University of Hong Kong, Hong Kong*

Andrea Huber Brösamle
*Helmholtz Center Munich, Germany*

Kazuhiro Ikenaka
*National Institute for Physiological Sciences, Japan*

Stephen Jackson
*University of Nottingham, UK and Korea University, South Korea*

Takeshi Kaneko
*Kyoto University, Japan*

Kenneth Kosik
*University of California, Santa Barbara, USA*

Klas Kullander
*Uppsala University, Sweden*

Jonathan Lee
*University of Birmingham, United Kingdom*

Edward Levin
*Duke University, USA*

Randolph Marshall
*Columbia University Medical Center, USA*

Cheryl McCormick
*Brock University, Canada*

Douglas Mennin
*Hunter College, USA*

Martin M. Mortazavi
*University of Alabama, Birmingham, USA*

Declan Murphy
*Kings College London, United Kingdom*

Heinz Reichmann
*University of Dresden, Germany*

Richard Rende
*Brown University, USA*

Giacomo Rizzolatti
*University of Parma, Italy*

Fred Sabb
*University of California, Los Angeles, USA*

Veronika Skvortsova
*Russian State Medical University, Russian Federation*

Kai-Christian Sonntag
*Harvard Medical School, McLean Hospital, USA*

Takashi Suzuki
*Max Planck Institute of Neurobiology, Germany*

Tanya N. Turan
*Medical University of South Carolina, USA*

Hartmut Wekerle
*Max Planck Institute of Neurobiology, Germany*

***Consulting Editors***

Alexander Bystritsky
*University of California, Los Angeles, USA*

Wesley Thompson
*University of California, San Diego, USA*
